# Tissue Specificity of Decellularized Rhesus Monkey Kidney and Lung Scaffolds

**DOI:** 10.1371/journal.pone.0064134

**Published:** 2013-05-22

**Authors:** Karina H. Nakayama, C. Chang I. Lee, Cynthia A. Batchelder, Alice F. Tarantal

**Affiliations:** 1 California National Primate Research Center, University of California Davis, Davis, California, United States of America; 2 Departments of Pediatrics and Cell Biology and Human Anatomy, School of Medicine, University of California Davis, Davis, California, United States of America; The University of Tennessee Health Science Center, United States of America

## Abstract

Initial steps in establishing an optimal strategy for functional bioengineered tissues is generation of three-dimensional constructs containing cells with the appropriate organization and phenotype. To effectively utilize rhesus monkey decellularized kidney scaffolds, these studies evaluated two key parameters: (1) residual scaffold components after decellularization including proteomics analysis, and (2) the use of undifferentiated human embryonic stem cells (hESCs) for recellularization in order to explore cellular differentiation in a tissue-specific manner. Sections of kidney and lung were selected for a comparative evaluation because of their similar pattern of organogenesis. Proteomics analysis revealed the presence of growth factors and antimicrobial proteins as well as stress proteins and complement components. Immunohistochemistry of recellularized kidney scaffolds showed the generation of Cytokeratin+ epithelial tubule phenotypes throughout the scaffold that demonstrated a statistically significant increase in expression of kidney-associated genes compared to baseline hESC gene expression. Recellularization of lung scaffolds showed that cells lined the alveolar spaces and demonstrated statistically significant upregulation of key lung-associated genes. However, overall expression of kidney and lung-associated markers was not statistically different when the kidney and lung recellularized scaffolds were compared. These results suggest that decellularized scaffolds have an intrinsic spatial ability to influence hESC differentiation by physically shaping cells into tissue-appropriate structures and phenotypes, and that additional approaches may be needed to ensure consistent recellularization throughout the matrix.

## Introduction

There is an ongoing interest in using native extracellular matrix (ECM) scaffolds derived from decellularized tissues as a potential method for repair of organs damaged by disease. Although results are promising, the clinical outcome of recent studies, such as those involving transplantation of tissue engineered decellularized airways, present issues such as the biomechanical collapse of the graft [1]–[3]. These findings indicate a more exhaustive *in vitro* understanding of the components of decellularized tissues is required before translational applications can be fully realized and achieved. For example, there is a need to explore fundamental questions related to scaffold properties including the contents that remain after the decellularization process and the tissue-specific properties that may potentiate lineage specification during recellularization. Differences in ECM composition, morphology, and structural properties are also known to impact scaffold physical properties and biological outcomes [4]–[6]. ECM proteins provide physical biomechanical structure and interactive ligand-mediated feedback to guide and alter the fate of cells. These features must be carefully considered in order to better understand and effectively utilize the native ECM architecture provided by decellularized scaffolds for tissue repair.

Studies by our group have demonstrated that rhesus monkey kidneys of all age groups (fetal to adult) can be decellularized to provide a natural ECM with preserved structural and biological properties [7]. These scaffolds were shown to support migration of renal cells from kidney explants in an age-dependent manner, and elongation of tubules that was enhanced with the addition of cytokines [8]. The first goal of the studies described herein was to characterize the residual contents of kidney and lung scaffolds including DNA/RNA, Major Histocompatibility Complex (MHC) antigens, cellular markers, growth factors, and proteins. Kidney and lung were selected for a comparative evaluation because of their similar pattern of organogenesis, which is driven by reciprocal mesenchymal-to-epithelial interactions that lead to branching morphogenesis. The second goal was to determine the tissue specificity of the scaffolds by comparing recellularization efficiency using human embryonic stem cells (hESCs). Thus, two central issues were explored: detection of the residual components remaining in decellularized tissue scaffolds which could impact the recellularization process and ultimately transplantation, and if decellularized kidney and lung scaffolds exhibit tissue-specific properties and the ability to drive hESC differentiation in a tissue specific manner.

## Materials and Methods

### Ethics Statement and Tissue Collection

All animal protocols were approved prior to implementation by the Institutional Animal Care and Use Committee (IACUC) at the University of California, Davis, and all procedures conformed to the requirements of the Animal Welfare Act. Activities related to animal care including housing, feeding, and environmental enrichment were performed in accordance with IACUC-approved standard operating procedures (SOPs) at the California National Primate Research Center (http://www.cnprc.ucdavis.edu). Euthanasia was consistent with the recommendations of the American Veterinary Medical Association (AVMA) Guidelines on Euthanasia and Primate Center SOPs (overdose of pentobarbital). Tissue collection was performed using established methods and included juvenile (1–3 years) kidneys (transverse sections) (N = 3) and caudal lung lobes (sagittal sections) (N = 3) from rhesus monkeys (*Macaca mulatta*) as previously described [7]. All tissues were placed in Roswell Park Memorial Institute medium (RPMI, Life Technologies, Grand Island, NY) upon collection with processing conducted at the time of tissue harvest.

### Decellularization

Decellularization of tissue sections was performed using established protocols [8]. Briefly, kidney transverse sections were washed with phosphate buffered saline (PBS, Life Technologies) followed by a decellularization solution of 1% (v/v) sodium dodecyl sulfate (SDS, Life Technologies) or 0.1% (v/v) for lung sections diluted in UltraPure™ sterile distilled water (Life Technologies) at 4°C. Lung decellularization was optimized with detergents Triton X-100 and SDS and with three detergent concentrations 0.01%, 0.1%, and 1% v/v all carried out at 4°C. The solution was changed 8 h after initial tissue harvest and then every 48 h until the tissue was transparent (approximately 10–14 days for kidney sections and 20–24 days for lung sections). Decellularized scaffolds were gently washed with PBS and stored in 1% (v/v) penicillin-streptomycin (pen-strep, Life Technologies) in PBS at 4°C until use (0–2 months maximum).

### Proteomics

Protein-works Protein Profiling service with 40 fractions per sample was performed by MS Bioworks LLC (Ann Arbor, MI) on one decellularized kidney scaffold biopsy and one lung scaffold biopsy. The punch biopsy was collected from a transverse kidney section or sagittal caudal lung lobe section using a sterile 8 mm hollow Biopunch® (Healthlink, Jacksonville, FL). Samples were extracted by mechanical tissue disruption in a Bullet Blender (Next Advance, Averill Park, NY) in 50 mM Tris-HCl, pH 8.0, 150 mM NaCl, 0.1% SDS, 0.05% ProteaseMax (Promega, Madison, WI), 1X Complete Protease Inhibitor (Roche, Pleasanton, CA), and 1X PhoStop phosphatase inhibitor (Roche). Cell debris was pelleted via centrifugation and the supernatant was quantified using a Qubit fluorometer (Life Technologies). Each sample (20 µg) was loaded onto a 4–12% SDS-PAGE gel (Novex, Life Technologies). The gel was excised into 40 segments per lane and gel slices were processed using a robot (ProGest, DigiLab, Holliston, MA) for the following processing cycle: 25 mM ammonium bicarbonate wash, acetonitrile wash reduction with 10 mM dithiothreitol at 60°C, alkylation with 50 mM iodoacetamide at room temperature, digestion with trypsin (Promega) at 37°C for 4 h, and quenching with formic acid. The supernatant was analyzed directly without further processing.

Each gel digest was analyzed by nano liquid chromatography and tandem mass spectrometry (LC/MS/MS) with a Waters NanoAcquity HPLC system interfaced to a ThermoFisher Orbitrap Velos Pro Peptides (Thermo Fisher Scientific, Waltham, MA) and were loaded on a trapping column and eluted over a 75 µm analytical column at 350 nL/min; both columns were packed with Jupiter Proteo resin (Phenomenex, Torrance, CA). The mass spectrometer was operated in data-dependent mode, with MS performed in the Orbitrap at 60,000 full width at half maximum (FWHM) resolution and MS/MS performed in the linear trap quadrupole (LTQ). The 15 most abundant ions were selected for MS/MS.

Data were searched using a local copy of Mascot with the following parameters: Enzyme: Trypsin; Database 1: Uniprot *M. mulatta* (concatenated forward and reverse plus common contaminants); Database 2: NCBI *M. mulatta* (concatenated forward and reverse plus common contaminants); Fixed modification: Carbamidomethyl (C); Variable modifications: Oxidation (M), Acetyl (Protein N-term), Deamidation (NQ), Pyro-Glu (N-term Q); Mass values: Monoisotopic; Peptide Mass Tolerance: 10 ppm; Fragment Mass Tolerance: 0.8 Da; Max Missed Cleavages: 2.

Mascot DAT files were parsed into Scaffold software (Proteome Software, Inc., Portland, OR) for validation, filtering, and to create a non-redundant list per sample. Data were filtered using a minimum protein value of 90%, a minimum peptide value of 50% (Prophet scores), and requiring at least two unique peptides per protein.

Data were searched against both NCBI *M. mulatta* and Uniprot *M. mulatta* databases. Peptides belonging to a given protein were normalized to the corresponding molecular weight. The abundance of each protein in the scaffold was assessed based on the percentage each occupied of the total normalized peptide spectra.

### Recellularization with hESCs

Prior to recellularization, scaffolds were washed overnight in sterile filtered (0.22 µm pore size, Millipore, Billerica, MA) 70% (v/v) ethanol in super-Q water at room temperature on a Dynal tube rotator (Dynal, Inc., New Hyde Park, NY) at 25 revolutions/min and rehydrated with several 24 h washes of PBS with 1% pen-strep.

**Figure 1:**
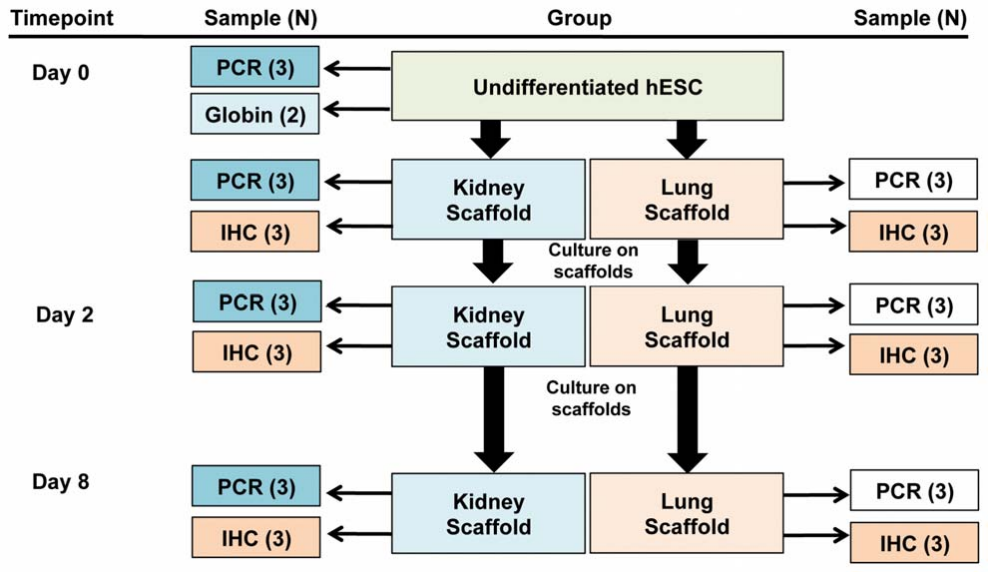
Sample collection. Immunohistochemistry (IHC), human embryonic stem cells (hESC).

hESCs (WA09 [H9]; NIH number 0062; passage 36) were cultured on irradiated mouse embryonic feeders and expanded from an established bank that was characterized as described previously for pluripotency markers and shown to spontaneously differentiate via embryoid bodies towards ectoderm, endoderm, and mesoderm [9]. Samples of undifferentiated cells (Day 0) were obtained for quantitative PCR (qPCR) and stored in RNeasy Lysis Buffer (Buffer RLT, Qiagen, Valencia, CA) with 1% 2-Mercaptoethanol at ≤-20°C until processed. Samples were also collected in PBS (1×10^6^ cells) to develop a standard curve to equate ε-globin (housekeeping gene) copy number with cell numbers. Juvenile kidney (N = 3) and lung (N = 3) scaffolds were cut into cylindrical biopsies (8 mm diameter) and seeded with approximately 5×10^5^ undifferentiated hESCs on Day 0. Non-seeded decellularized kidney and lung scaffolds were also collected on Day 0 for histological analysis. Each seeded scaffold was suspended on PET track-etched membrane cell culture inserts (1 cm diameter, 1.0 µm pore size, BD Biosciences, San Jose, CA). Once seeded, all scaffolds were cultured in Dulbecco's Modified Eagle's Medium high glucose with 10% fetal bovine serum (FBS), 1% pen-strep, and 1% L-glutamine at 37°C and 5% CO_2_. Two days after cells were seeded onto the scaffolds, each scaffold was gently transferred to new culture inserts to quantify non-adherent cells remaining in the original inserts with a hemacytometer and viability assessed with trypan blue. A reference Day 2 scaffold was collected from each group and stored at 4°C in 1 ml RNA*later*® stabilization solution (Ambion, Foster City, CA) for 48 h and then at ≤-80°C until processed for qPCR. On Day 8 scaffolds were collected for morphological analysis by fixing in 10% phosphate buffered formalin (Thermo Fisher Scientific, Waltham, MA) and embedded in paraffin or collected for qPCR (stored at 4°C in 1 ml RNA*later*® for 48 h and then at ≤-80°C until processed) (Figure 1).

Approximately 5×10^5^ hESCs were seeded onto Matrigel™ (BD Biosciences; in a 50∶50 dilution of Matrigel™ to medium) or were cultured in low adhesion plates to form embryoid bodies. Matrigel™ controls were allowed to form a semi-solid gel in the culture inserts to serve as three-dimensional (3D) ECM controls. Matrigel™ and embryoid body control samples (N = 3) were collected on day 8 of culture for immunohistochemistry (IHC) by dissolving Matrigel™ with cold PBS, followed by centrifugation at 1,500 revolutions per min (rpm) for 5 min at 4°C. Cell pellets for IHC were gently fixed in 10% formalin for 48 h and embedded in paraffin.

hESCs were cultured in medium for embryoid body formation with solutions of 0%, 0.0001%, 0.001%, 0.01%, or 0.1% SDS (v/v) in low-adhesion plates for 8 days, with medium changes every 48 h. At the end of the culture period embryoid bodies were collected, washed with PBS, and centrifuged at 1,500 rpm for 5 min to form a pellet. Pellets were fixed in 10% formalin for 48 h and embedded in paraffin for IHC.

To assess the effects of soluble molecules released from empty scaffolds on cell surfactant protein expression, empty decellularized juvenile kidney scaffolds (N = 3) were incubated with medium for 8 days. Every 24 h, the incubated medium was transferred from the empty scaffold culture to low adhesion plates containing hESCs for embryoid body formation. After 8 days, scaffolds and pellets were collected and fixed in 10% formalin for 48 h and embedded in paraffin for IHC.

### Morphology

Morphology and scaffold recellularization were assessed by serial sectioning (5–6 µm) of the entire recellularized paraffin-embedded scaffold. Hematoxylin and Eosin (H&E) staining and IHC was performed as described [8]. Briefly, slides were rehydrated in xylene followed by graded concentrations of ethanol and washed in PBS before heat-mediated antigen retrieval in citrate buffer (pH 6, Life Technologies). Slides were cooled in decreasing concentrations of warm citrate buffer in PBS followed by incubation with Background Sniper (BioCare Medical, Concord, CA) which was added to each slide for 15 min. Slides were washed twice with PBS followed by incubation for 1 h with blocking buffer (1% bovine serum albumin [BSA], 0.1% fish skin gelatin, 0.1% Triton X-100, 0.05% Tween-20) with 2% goat serum (Sigma, St. Louis, MO). After two washes with PBS, primary antibody diluted in primary antibody buffer (1% BSA, 0.1% fish skin gelatin) was incubated with slides overnight in a humidified chamber at 4°C. Primary antibodies used are listed in Table S1 and included human leukocyte antigen-DR (HLA-DR, clone LN3, Life Technologies) diluted 1∶50, Surfactant Protein-B (SP-B, polyclonal, Abcam, Cambridge, MA) diluted 1∶1000, Thyroid-specific transcription factor/NK2 homeobox 1 (TTF1, clone 8G7G3/1, Abcam) diluted 1∶1000, Vimentin (clone V6389, Sigma) diluted 1∶100, wide spectrum Cytokeratin (polyclonal, Abcam) diluted 1∶50, and Mouse IgG1 (Dako, Carpinteria, CA) and rabbit IgG (Life Technologies) served as isotype controls. Slides were washed twice with PBS for 5 min and incubated with secondary antibody for 1 h in the dark at room temperature. Secondary antibodies used were Alexa Fluor® 488 goat anti-mouse (Life Technologies) and Alexa Fluor® 594 goat anti-rabbit (Life Technologies) diluted 1∶200 in fluorescence antibody diluent (BioCare Medical). After washing twice with PBS, slides were mounted with ProLong Gold® antifade reagent with 4′,6-diamidino-2-phenylindole (DAPI) (Life Technologies) and a coverslip placed.

3,3′-Diaminobenzidine (DAB) staining was performed for HLA-E to assess MHC class I antigens in the decellularized scaffolds and for SP-B and SP-C to further assess surfactant expression in the recellularized kidney and lung scaffolds. Staining was performed using Dako EnVision+System-HRP (DAB) kit (Dako) according to the manufacturer’s instructions. Primary antibodies used were HLA-E (clone MEM-E02, Thermo Fisher Scientific) diluted 1∶100, SP-B diluted 1∶1000, and SP-C (polyclonal, Abcam) diluted 1∶500 in primary antibody buffer with 5% goat serum. Slides were mounted with Cytoseal 60 mounting medium (Andwin Scientific, Tryon, NC).

### Quantitative RT-PCR for Kidney and Lung Genes

RT^2^ Profiler™ PCR (Qiagen) array 96-well plates assessed gene expression of 11 kidney-associated genes or 11 lung-associated genes (Table S2 and Table S3, respectively). Total DNA and RNA were extracted from cells preserved in Buffer RLT at ≤-20°C and from scaffolds preserved in RNA*later*® solution at ≤-80°C using the AllPrep DNA/RNA Mini kit (Qiagen) following the manufacturer's instructions. Scaffolds were processed in multiple sections weighing <30 µg each to ensure efficient isolation of DNA and RNA.

cDNA was synthesized using the RT^2^ First Strand Kit (Qiagen) according to the manufacturer’s instructions. The RT^2^ RNA QC PCR array (Qiagen) was used to assess RNA quality prior to gene expression analysis. Real-time PCR reactions (25 µl total volume) were carried out in custom array plates with RT^2^ SYBR® Green ROX™ qPCR Mastermix (Qiagen) following instructions provided by the manufacturer using a 7900® ABI Sequence Detection System (Applied Biosystems, Foster City, CA). The PCR protocol consisted of one cycle of 10 min at 95°C, followed by 40 cycles of 15 s at 95°C, and 1 min at 60°C. cDNA from three samples of undifferentiated hESCs were used as the calibrator.

RNA expression was quantified according to the Comparative *C*_T_ method described in User Bulletin #2 (Applied Biosystems, Foster City, CA; updated 2001) relative to the housekeeping gene hypoxanthine phosphoribosyltransferase (HPRT) [9]. Positive and negative controls (water) were included in each run. The results were calculated as relative transcription or the *n*-fold difference relative to a calibrator cDNA from three samples of undifferentiated hESCs.

The number of cells seeded per scaffold was quantified by RT-PCR analysis of ε-globin after generation of a standard curve to equate copy number with cell number [9]. qPCR reactions were run in duplicate in 25 µl reaction volumes containing TaqMan Universal PCR Master Mix (Applied Biosystems) with 400 nM of forward and reverse primers and 100 nM probe. The PCR protocol consisted of one cycle of 2 min at 50°C, 10 min at 95°C, followed by 40 cycles at 15 s at 95°C, and 60 s at 60°C. Variations in scaffold size were normalized by comparison of ε-globin copy number per mg of tissue.

### Statistical Analysis

All data are shown as mean with the standard error of the mean (SEM). Statistical analysis was performed using a two tailed student’s t-Test with *p* ≤ 0.05 considered statistically significant.

## Results

### Decellularized Kidney and Lung Scaffold Characterization

Our prior studies have shown intact cells are removed by the decellularization process as determined by the absence of H&E and DAPI staining of cell nuclei in decellularized kidney scaffolds [7]. The quantity of residual DNA in the scaffolds compared to fresh tissue was determined based on copies of the housekeeping gene, ε-globin, by qPCR. These studies indicated that 95.6±0.6% of DNA was removed in decellularized scaffolds. Thus, although the majority of DNA was removed by decellularization, scaffolds did retain a small quantity of residual DNA.

**Figure 2:**
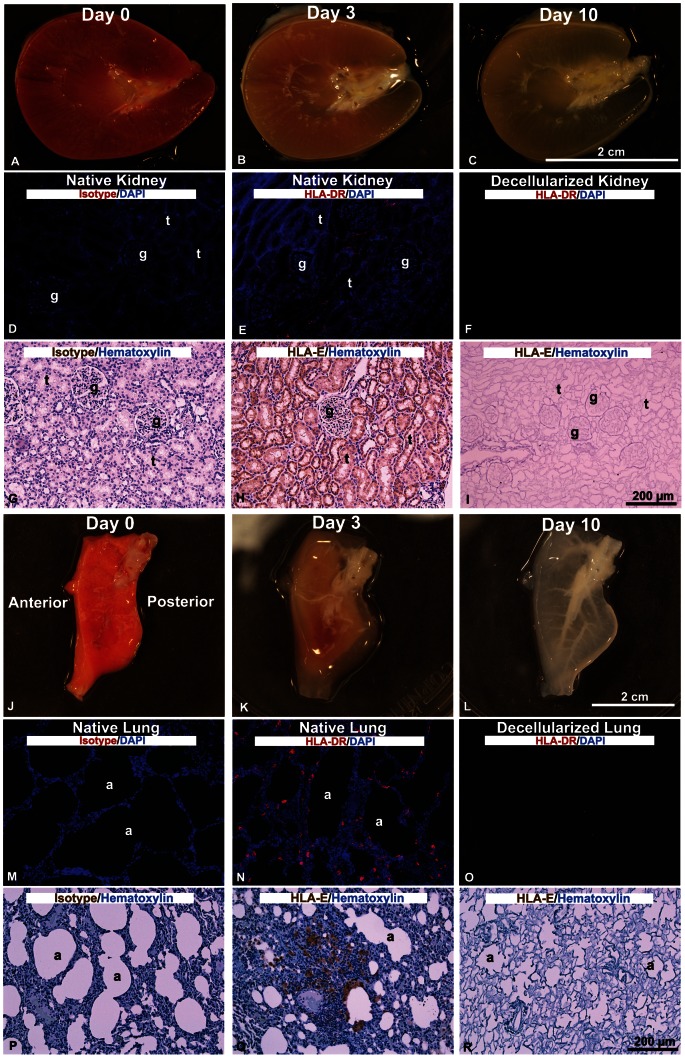
Decellularization of rhesus monkey kidney and lung sections. Decellularization of kidney transverse sections with 1% SDS (**A**–**C**) over 10 days and IHC for MHC class I (HLA-E) and MHC class II (HLA-DR) markers in the native and decellularized kidney scaffold (**D**–**I**). Decellularization of sagittal caudal lung lobe sections with 0.1% SDS (**J**–**L**) over 10 days and IHC of HLA-E and HLA-DR markers in the native and decellularized lung scaffold (**M**–**R**). 4′,6-diamidino-2-phenylindole (DAPI) for cell nuclei.

Optimization of decellularization demonstrated that 1% SDS for kidney and 0.1% SDS for lung yielded scaffolds with preserved ECM structure while removing cells including MHC class I (HLA-E) and II (HLA-DR) antigens as assessed by IHC (Figure 2). Absence of DAPI and hematoxylin staining in the kidney and lung scaffolds demonstrated cell nuclei were removed. Immunofluorescent staining for HLA-DR and DAB staining for HLA-E was evident in the native kidney and lung and showed no staining in decellularized kidney and lung scaffolds confirming removal of both MHC antigens.

### Comparison of Kidney and Lung Scaffold Composition

**Figure 3:**
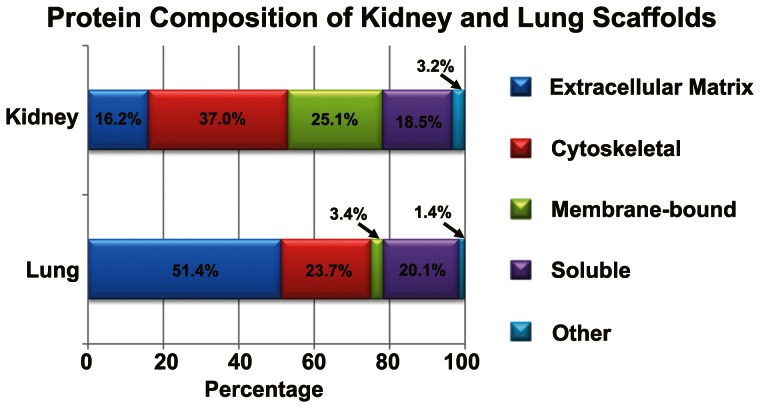
Protein composition of kidney and lung scaffolds. Compositional breakdown of proteins found in kidney and lung.

Proteomics analysis identified 439 proteins in the kidney scaffold and 282 proteins in the lung scaffold (including known contaminants) with a false discovery rate of 0.2% for the kidney and 3.1% for the lung. After removal of known contaminants, there were 220 proteins detected in the kidney scaffold that were not present in the lung scaffold, 130 proteins expressed in the lung scaffold that were not present in the kidney scaffold, and 110 proteins expressed in both scaffolds. The top 100 most abundant proteins in each scaffold were grouped according to function (Figure 3). The most abundant ECM proteins found in the kidney were Annexin A2 (basement membrane; possible roles in heat-stress response, calcium binding, angiogenesis), Fibrillin 1 (glycoprotein; fibrillin-1-containing microfibrils provide long-term force bearing structural support), and Collagen alpha-1(IV) chain (glycoprotein; role in cell adhesion, platelet-derived growth factor binding) [10]. The most abundant ECM proteins found in the lung decellularized scaffold were Periostin (secreted protein; role in cell adhesion and heparan binding), Decorin (secreted protein; role in type I and II Collagen, Fibronectin, and Transforming Growth Factor-β [TGF-β, binding; matrix assembly]) [10], and Collagen alpha-1(IV). Non-ECM proteins accounted for approximately 80% of the proteins found in the kidney scaffold and approximately 50% of the proteins found in the lung scaffold.

The scaffolds retained many proteins that have been viewed as potentially beneficial [11] including a wide array of ECM proteins, keratins, antimicrobial proteins such as Dermcidin and Defensin, and growth factors including TGF-β, Epidermal Growth Factor-7 (EGF-7), and Heparin-binding Growth Factor-2 (Table S4). Also noted were a number of proteins with the potential to induce an adverse response by cells to the scaffold environment including the presence of several complement components (CC6–CC9), stress proteins (Heat shock cognate 71 kDa protein, Heat shock protein β-1), and apoptosis-inducing proteins. Uromodulin (UMOD), a renal tubule marker, was noted in very low quantity (represented <0.03% total protein) in the kidney scaffold, and a lung pneumocyte marker, von Willebrand factor (VWF), was noted in low quantity (represented <0.15% total protein) in the lung scaffold. Neither HLA-E nor HLA-DR were detected. The common housekeeping gene, Eukaryotic translation elongation factor 1-alpha (EF1-α), was also present but not HPRT. Therefore, HPRT was used for the internal control for gene expression.

Residual gene expression for kidney and lung-associated genes was assessed in decellularized kidney and lung scaffolds; however, the residual expression of the housekeeping gene, HPRT, was extremely low (*C*_T_ >39). These findings suggest that cellular mRNA, including common housekeeping genes, were effectively removed by the decellularization process and that residual gene expression in decellularized scaffolds would not interfere with qPCR results.

### Comparison of Recellularization: Gene Expression

**Figure 4:**
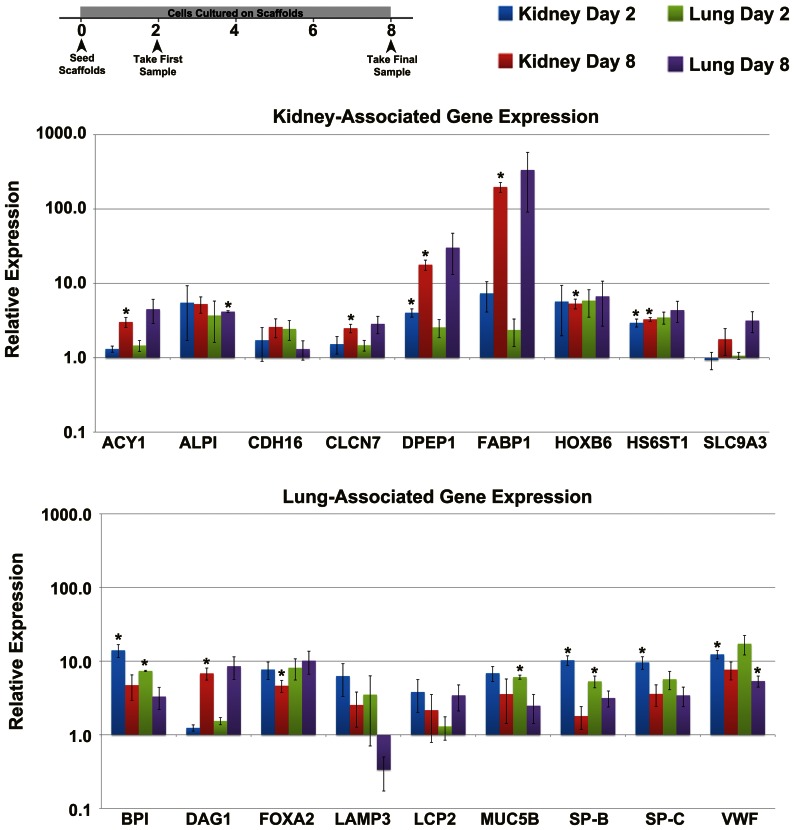
Gene expression of recellularized kidney and lung scaffolds. Timeline indicates timing of sample collection. Gene expression of kidney-associated genes: Aminoacylase 1 (ACY1), Intestinal alkaline phosphatase (ALPI), Cadherin-16 (CDH16), Chloride Channel protein (CLCN7), Dipeptidase 1 renal (DPEP1), Fatty acid binding protein 1 (FABP1), Homeobox protein B6 (HOXB6), Heparan sulfate 6-O-sulfotransferase 1 (HS6T1), and Solute carrier family 9 member 3 (SLC9A3) (**A**), and lung-associated genes: Bactericidal/permeability-increasing protein (BPI), Dystroglycan 1 (DAG1), Forkhead box A2 (FOXA2), Lysosomal-associated membrane protein 3 (LAMP3), Lymphocyte cytosolic protein 2 (LCP2), Mucin 5B (MUC5B), Surfactant protein-B (SP-B), SP-C, and Von Willebrand factor (VWF) (**B**) in the recellularized kidney and lung scaffolds after 2 and 8 days in culture ± SEM. Statistically significant differences in expression compared to undifferentiated hESCs from Day 0 are indicated by asterisks (*p ≤ 0.05).

Undifferentiated hESCs were seeded onto juvenile decellularized kidney or lung scaffolds. Gene expression of kidney or lung-associated genes was assessed by qPCR. Compared to baseline gene expression of undifferentiated hESCs (Day 0), expression levels of tubule markers, Dipeptidase 1 renal (DPEP1) and Heparan sulfate 6-O-sulfotransferase 1 (HS6T1), significantly increased after 2 days of culture on kidney scaffolds and remained elevated throughout the 8 days of culture. Other genes upregulated by culture on kidney scaffolds on Day 8 included fetal kidney tubule makers, Chloride Channel protein (CLCN7), and Homeobox protein B6 (HOXB6), as well as two markers of proximal tubules, Aminoacylase 1 (ACY1), and Fatty acid binding protein 1 (FABP1) (Figure 4A). Although a trend of increased expression of kidney genes on the lung scaffolds was observed, only an increase in Intestinal alkaline phosphatase (ALPI), another proximal tubule marker, was statistically significant compared to Day 0. Taken together, these data confirmed a greater number of kidney genes upregulated in cells cultured on kidney scaffolds than on lung scaffolds.

Several lung-associated genes including Bactericidal/permeability-increasing protein (BPI), which is expressed by respiratory epithelial cells of the bronchus and nasopharynx, SP-B, SP-C, and VWF, which are expressed by pneumocytes, were transiently elevated in kidney scaffolds by Day 2 but returned to baseline by Day 8 (Figure 4B). Similarly, BPI, Mucin 5B (MUC5B), another marker of respiratory epithelial cells, and SP-B were upregulated on the lung scaffolds after 2 days in culture but by 8 days only VWF demonstrated increased gene expression compared to Day 0. Based on these data, pneumocyte and respiratory epithelial genes were transiently upregulated after initial introduction of cells to both the kidney and lung scaffolds but statistical significance was not apparent after further culture.

Expression of kidney genes, UMOD (Tamm-Horsfall glycoprotein) and Solute carrier family 12 (SLC12A), as well as the lung genes, Clara Cell specific protein (Uteroglobin, SCGB1A1) and TTF1/NK2 homeobox 1 (TTF1/NKX2.1), were not detected in cells cultured on either kidney or lung scaffolds. Compared to Day 0 undifferentiated hESCs, the cells cultured on kidney and lung scaffolds showed significant differences in tissue-specific gene expression over time, indicating that the scaffold was able to partially stimulate differentiation of hESCs to express tissue-specific markers. However, when gene expression levels of the differentiating cells on kidney versus lung scaffolds were compared on the same day, no statistically significant differences were found. These findings suggest that the gene expression response of hESCs to the scaffold ECM is similar for kidney and lung scaffolds despite statistical significance when compared to undifferentiated hESCs. Hence, the scaffolds were able to drive differentiation of hESCs to express kidney and lung genes, but no statistically significant difference in the gene expression patterns between hESCs seeded on kidney versus lung scaffolds were detected.

### Comparison of Recellularization: Morphology

**Figure 5:**
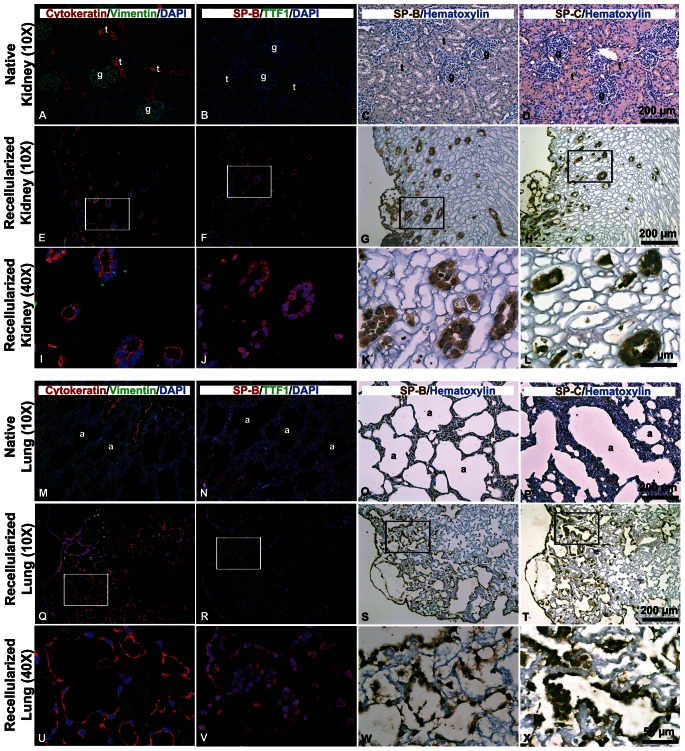
IHC of decellularized kidney and lung scaffolds recellularized with undifferentiated hESCs. Staining is shown in the native kidney (**A**–**D**) and recellularized kidney scaffold (**E**–**H**, 10X; **I**–**L**, 40X), native lung (**M**–**P**), and recellularized lung scaffold (**Q**–**T**, 10X; **U**–**X**, 40X). Staining was performed for Cytokeratin and Vimentin (**A**, **E**, **I**, **M**, **Q**, **U**), SP-B and Thyroid-specific transcription factor/NK2 homeobox 1 (TTF1) (**B**, **F**, **J**, **N**, **R**, **V**), SP-B with DAB (**C**, **G**, **K**, **O**, **S**, **W**), or SP-C with DAB (**D**, **H**, **L**, **P**, **T**, **X**). The white or black box in the 10X image highlights the region magnified in the higher magnification 40X image directly below; glomeruli (g), tubules (t), alveolus (a). DAPI = cell nuclei.

Cells in the kidney scaffold formed thick symmetrical epithelial (Cytokeratin+) tubules, whereas cells in the lung scaffolds lined the alveolar spaces and formed thin networks of surfactant protein expressing cells (Figure 5). Despite different morphology and spatial organization, cells in both scaffolds expressed Cytokeratin, SP-B, and SP-C.

**Figure 6:**
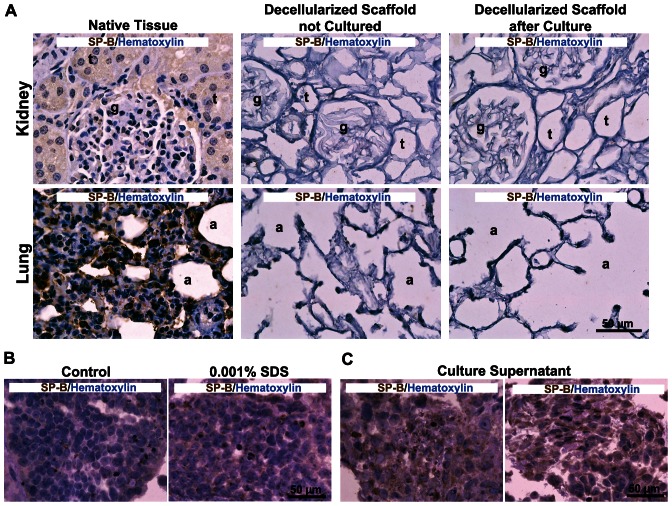
SP-B staining of decellularized scaffolds and embryoid bodies. IHC for SP-B on decellularized kidney and lung scaffolds before culture and after 8 days of culture in medium (**A**). Fresh kidney and lung stained for SP-B were included for negative and positive controls; glomeruli (g), tubules (t), alveolus (a). SP-B staining of embryoid bodies cultured for 8 days in 0% (Control) and 0.001% SDS in medium (**B**). SP-B staining of embryoid bodies cultured for 8 days in supernatant from incubated decellularized kidney scaffolds (**C**).

To determine if the observed expression of surfactant proteins was confounded by residual staining of proteins remaining in the decellularized scaffold or was due to a response to a non-specific 3D ECM environment, IHC was performed on kidney and lung decellularized scaffolds (non-seeded) and hESCs cultured in Matrigel™, respectively. IHC performed on decellularized scaffolds prior to as well as following incubation with medium did not stain for surfactant proteins (Figure 6A). These findings suggest that surfactant protein is not residually expressed in the scaffold nor acquired by the scaffold after culture. Cells in the Matrigel™ controls did not express SP-B but weakly expressed SP-C, suggesting a minor degree of non-specific differentiation of hESCs in response to a non-tissue-specific ECM-rich environment.

To examine the possible contribution of residual soluble molecules in the decellularized scaffolds on the stimulation of surfactant protein expression, embryoid bodies were exposed to various concentrations of SDS in culture medium or the supernatant from non-seeded decellularized scaffolds incubated in medium. Surfactant protein expression in embryoid bodies was not affected by the addition of SDS to the medium (Figure 6B). Staining of cells cultured in 0.00001%, 0.0001%, or 0.001% SDS appeared similar to control with 0% SDS (medium alone). Cells cultured in medium with SDS concentrations of 0.01% or greater displayed membrane lysis and could not be collected at the end of the 8-day culture period. In addition, control embryoid bodies were Vimentin+ (mesoderm and parietal endoderm) and did not express Cytokeratin, SP-B, or SP-C. Interestingly, culture of embryoid bodies with supernatants from empty scaffolds induced mild diffuse surfactant protein expression (Figure 6C). These findings suggest that soluble molecules released from cultured scaffolds may partially be responsible for stimulating surfactant expression, likely in a dose-dependent manner and dictated by the amount of the molecules remaining in the original decellularized scaffold.

## Discussion and Conclusions

Decellularized tissue scaffolds provide a platform for developing functional tissue replacements by offering mechanical, structural, and biological properties similar to the native tissues from which they were derived. The first goal of the current study was to characterize the residual contents of kidney and lung scaffolds including DNA/RNA, MHC antigens, cellular markers, growth factors, and proteins to better understand and utilize the scaffolds for translational therapies. Kidney and lung were selected for a comparative evaluation because of their similar pattern of organogenesis, which is driven by reciprocal mesenchymal-to-epithelial interactions that lead to branching morphogenesis. Development of the kidney and lung depends on many of the same cytokines such as retinoids and bone morphogenic proteins [12]. These two organs are highly vascularized and have complex branching networks of specialized cells. Moreover, differences in developmental transcription factors enabled evaluation of tissue-specific expression of genes that are not shared between the kidney and lung during development. In addition, physical differences in composition may aid in determining how the kidney versus lung must be decellularized, and the approaches necessary for effective recellularization.

It has been suggested that decellularized scaffolds retain cytokines that are beneficial for scaffold recellularization and host integration. Decellularized tracheal and brain matrices maintain expression of basic fibroblast growth factor-2 (FGF-2) and TGF-β, respectively. The presence of residual cytokines in decellularized matrices was sufficient to provide pro-angiogenic properties using the chicken embryo chorioallantoic membrane [13]–[15]. Similar to studies with tracheal and brain matrices, and through proteomics analysis, the current study revealed the presence of several residual growth factors in the kidney and lung scaffolds that may be beneficial in scaffold recellularization. Insulin-like growth factor binding protein-7, which plays a role in cellular proliferation and vessel stabilization [16], was detected in the kidney scaffold. In addition, both kidney and lung scaffolds contained EGF-7 precursor, which plays a role in vascular tubulogenesis and protects endothelial cells from hypoxia-induced apoptosis [17]. EGF-7 has also been implicated in kidney development and shown to enable transformation of mesenchymal cells to stromal (interstitial) cells [18]. Another factor present in both kidney and lung scaffolds was TGF-β, which has been shown with FGF-2 to induce nephrogenesis through a Wnt-dependent pathway and develop the polarized tubular epithelia of the nephron [19], [20]. Heparin-binding growth factor-2/FGF-2 was detected in the lung scaffold and is one of the most potent mitogens in stimulating lung epithelial cell proliferation and is an inducer of surfactant protein genes [21]. The presence of these growth factors may be sufficient to potentiate cell infiltration and differentiation within the scaffold. Additional studies will be needed to assess the contribution of each growth factor to recellularization and determine whether the quantity present after decellularization is sufficient to induce a cellular response.

In addition, decellularized tissues were shown to retain antimicrobial proteins that may be beneficial for *in vitro* culture as well as downstream host integration. Potent antimicrobial and anti-fungal proteins such as Dermcidin-like protein found in kidney and lung scaffolds, and Defensin found in the lung, are consistent with findings by others who have demonstrated bacterial resistance of ECM-derived bladder and liver scaffolds [22].

These studies also highlight the importance of addressing the question of whether certain scaffold components and residual proteins may retain elements that could result in rejection post-transplantation. PCR revealed 96% DNA removal thus a small quantity of DNA remained emphasizing that additional steps such as enzymatic digestion, more vigorous mechanical agitation, or additional washes that affect pH or dielectric constants may be necessary to further enhance removal of residual DNA without damaging the ECM. Undigested DNA from dead cells and endogenous mitochondrial ’damage’-associated molecular patterns resulting from cellular trauma can cause activation of the innate immune response and elicit neutrophil-mediated organ injury creating a sepsis-like state [23], [24]. Beneficial as well as concerning outcomes have been reported for macrophage activation in response to abdominal wall implantation of decellularized dermal or bladder tissues [25]–[27]. Thus, the potential consequences of residual cellular materials remains a concern.

Although proteomics analysis was performed on one kidney and one lung sample, additional samples would likely still reveal that ECM proteins only make up part of the several hundred proteins that remain in decellularized scaffolds. These findings suggest that in studies that utilize decellularized tissues, the cells are interacting with and responding to more than just the ECM. It is likely that the combination of ECM proteins with residual growth factors and other soluble molecules that remain in the decellularized scaffolds are responsible for directing differentiation and organization of the cells. This may be useful in cases of successful recellularization; however, these residual proteins may also be the cause of challenges encountered with consistent recellularization throughout the scaffold. It is not known if the successful use of decellularized tissues is only due to the structural benefits of the ECM proteins or if it is the combinatorial effects of ECM with an array of residual growth factors and molecules. However, preservation of these additional factors may also mean retention of other detrimental proteins that will present challenges for translation. Future studies will be needed to evaluate the potential consequences of the residual non-ECM proteins in an *in vivo* environment, as well as additional decellularization strategies to enhance the removal of potentially immunogenic materials including more rigorous washing strategies to improve protein antigen removal [28].

Several studies have suggested that residual SDS may prevent cell infiltration due to cytotoxicity. The protonated form of an amino group on lysine, which is displayed on ECM proteins, forms a strong bond to the negatively charged SDS [29], [30]. This study exposed embryoid bodies to a wide range of concentrations of SDS in medium; ≥0.01% was shown to result in complete lysis of cells. Thus, assumptions can be made that the concentration of residual SDS in decellularized scaffolds is well below 0.01% since lysis was not observed in cells grown within the scaffolds.

A promising finding from this study is the ability of the scaffolds to physically shape tissue-appropriate structures. hESCs formed tubules in kidney scaffolds and the same hESC population differentiated into surfactant protein-secreting cells that organized around alveolar spaces in the lung scaffolds. The ECM composition and presentation of membrane-bound and soluble proteins differs for a renal tubule when compared to an alveolus; therefore, it is expected that different contact-dependent pathways may be stimulated in hESCs upon adhesion to different scaffolds that promote tissue-specific differentiation. Cells in both scaffolds expressed Cytokeratin, SP-B, and SP-C. Although additional analysis would need to be performed to clearly identify and characterize the differentiated cells within the scaffolds, Cytokeratin typically stains epithelial cells of the respiratory tract, including basal, ciliated, goblet, and alveolar cells [31]. In the native kidney, Cytokeratin stains the epithelial cells of the Loop of Henle and collecting ducts [32]. SP-B is expressed by pneumocytes in the native lung whereas SP-C is expressed by pneumocytes as well as macrophages in the lung; weak staining has been noted in some kidney tubules [33].

To further characterize the differentiation state of the cells in each tissue scaffold, a panel of genes highly expressed in either kidney or lung was examined by qPCR. Here, results revealed that hESCs seeded onto decellularized kidney scaffolds acquired a significant increase in the expression of six kidney-associated markers whereas hESCs seeded onto lung scaffolds only acquired expression of one kidney-associated marker (ALPI). In contrast, short-term expression of lung-associated genes was observed regardless of the tissue scaffold on which undifferentiated hESCs were cultured. When examined in a longitudinal manner that followed gene expression of the cells over the entire culture period (8 days), statistically significant changes in tissue-specific gene expression were noted for cells on both scaffolds collected on Day 8 compared to the Day 0 undifferentiated hESC starting population. However, cross-comparison of a given gene for cells in kidney or lung scaffolds at a single time point demonstrated no statistical significance between the two scaffolds. These findings suggest that the scaffolds influence hESC differentiation but do not have the capability of genotypic lineage specification based solely on gene response. Alternative culture conditions may be required to induce appropriate and sustained tissue-specific gene expression. Because of their mechanistic similarities during ontogeny, the window of time included for the current study (8 days) may have limited the ability to fully capture divergence in gene expression of hESCs in kidney and lung scaffolds.

To investigate the expression of surfactant proteins by cells in the recellularized kidneys, embryoid bodies were cultured in a range of SDS concentrations to assess the potential contribution of residual SDS on inducing expression of these proteins by cells. Although no correlation was found between increasing concentrations of SDS and surfactant protein expression, diffuse surfactant expression was noted for cells exposed to the culture supernatants of non-seeded decellularized kidney scaffolds. This finding suggests that a soluble factor in the empty decellularized scaffold is released into the medium during the culture period and is responsible for stimulating mild surfactant protein expression by embryoid bodies and likely the hESCs in the kidney and lung scaffolds as well. Additional proteomics analysis could be used to identify all soluble proteins that are released from the empty decellularized scaffolds while in culture. It is likely that one or more of these soluble molecules were responsible for the observed surfactant protein expression.

In summary, this study highlights the potential use of decellularized tissues as a potent spatial mitogen and provides new insights into tissue-specific properties that can be exploited to drive cells from a pluripotent state to formation of tissue-specific structures without the addition of exogenous growth factors. Proteomics analysis of kidney and lung decellularized scaffolds also showed the inevitable translational hurdles in the form of hundreds of non-ECM residual proteins contained within scaffolds. Overall, these findings have demonstrated the ability of scaffold ECM to guide the spatial organization of cells into tissue-specific structures, although additional approaches will be needed to ensure consistent and efficient recellularization throughout the scaffold.

## Supporting Information

Table S1 **Antibodies for Immunohistochemistry**
(PDF)

Table S2 **Kidney Genes for qPCR**
(PDF)

Table S3 **Lung Genes for qPCR**
(PDF)

Table S4 **Protein Composition of Kidney and Lung Scaffolds**
(PDF)
