# Supplementary material for: Tissue Specificity of Decellularized Rhesus Monkey Kidney and Lung Scaffolds
Source: PLoS One. 2013 May 22;8(5):e64134. doi: 10.1371/journal.pone.0064134 (PMC3661477; doi:10.1371/journal.pone.0064134)
Supplement: Table S1 — Antibodies for Immunohistochemistry (PDF) [file pone.0064134.s001.pdf]

Table S1. Antibodies for Immunohistochemistry

#	Marker Name	Symbol	Clone	Kidney Expression	Lung Expression	Method
1	Human Leukocyte Antigen-DR	HLA-DR	LN3	Glomeruli, tubules	Pneumocytes, macrophages, respiratory epithelial cells	IF
2	Human Leukocyte Antigen-E	HLA-E	MEM-E02	Glomeruli	Pneumocytes, macrophages	DAB
3	Surfactant Protein B	SP-B	Polyclonal Rabbit	N/A	Pneumocytes	IF/DAB
4	Surfactant Protein C	SP-C	Polyclonal Rabbit	Some tubules	Pneuomocyte, macrophages	DAB
5	Thyroid-specific transcription factor/ NK2 homeobox 1	TTF1/ NKX2.1	8G7G3/1	N/A	Endoderm, pneuomocytes, macrophages	IF
6	Vimentin	Vimentin	V6389	Glomeruli, some tubules	Pneumocytes, macrophages	IF
7	Wide spectrum Cytokeratin	Cytokeratin	Polyclonal Rabbit	Epithelial cells of the Loop of Henle, collecting ducts	Respiratory epithelial cells (basal, ciliated, goblet, alveolar cells)	IF

IF=Immunofluorescence staining, DAB=3,3'-Diaminobenzidine staining, N/A=not applicable
