# Supplementary material for: Tissue Specificity of Decellularized Rhesus Monkey Kidney and Lung Scaffolds
Source: PLoS One. 2013 May 22;8(5):e64134. doi: 10.1371/journal.pone.0064134 (PMC3661477; doi:10.1371/journal.pone.0064134)
Supplement: Table S2 — Kidney Genes for qPCR (PDF) [file pone.0064134.s002.pdf]

Table S2. Kidney Genes for qPCR

#	Gene Name	Symbol	RefSeq #	Kidney Expression	Lung Expression
1	Aminoacylase 1	ACY1	NM_000666	Proximal tubules	N/A
2	Cadherin-16 (Kidney specific cadherin)	CDH16	NM_004062	Distal convoluted tubules	N/A
3	Chloride Channel protein	CLCN-7	NM_001287	Fetal tubules, glomeruli	Pneumocytes, macrophages
4	Dipeptidase 1 renal	DPEP1	NM_004413	Tubules	N/A
5	Fatty acid binding protein 1, liver	FABP1	NM_001443	Proximal tubules	N/A
6	Heparan sulfate 6-O-sulfotransferase 1	HS6ST1	NM_004807	Subset of tubules	N/A
7	Homeobox protein B6 (HOX 2.2)	HOXB6	NM_018952	Fetal tubules, glomeruli	Pneumocytes, macrophages
8	Intestinal alkaline phosphatase	ALPI	NM_001631	Proximal tubules	N/A
9	Solute carrier family 9 member 3 (NHE3)	SLC9A3	NM_004174	Nephron	Nasopharynx epithelial cells
10	Solute carrier family 12, member 1	SLC12A1	NM_000338	Tubules	Epithelial cells in bronchus, macrophages
11	Uromodulin (Tamm-Horsfall glycoprotein)	UMOD	NM_003361	Thick ascending limb, distal convoluted tubules	N/A

N/A=not applicable
