# Supplementary material for: Tissue Specificity of Decellularized Rhesus Monkey Kidney and Lung Scaffolds
Source: PLoS One. 2013 May 22;8(5):e64134. doi: 10.1371/journal.pone.0064134 (PMC3661477; doi:10.1371/journal.pone.0064134)
Supplement: Table S3 — Lung Genes for qPCR (PDF) [file pone.0064134.s003.pdf]

Table S3. Lung Genes for qPCR

#	Gene Name	Symbol	RefSeq #	Kidney Expression	Lung Expression
1	Bactericidal/permeability -increasing protein	BPI	NM_001725	N/A	Epithelial cells of bronchus, nasopharynx
2	Clara Cell specific protein (Uteroglobin)	SCGB1A1	NM_003357	N/A	Epithelial cells of bronchus
3	Dystroglycan 1 (dystrophin-associated glycoprotein 1)	DAG1	NM_004393	Tubules	Pneumocytes, macrophages
4	Hepatocyte nuclear factor-3 beta (forkhead box A2)	FOXA2	NM_021784	N/A	Epithelial cells, pneumocytes, macrophages
5	Lymphocyte cytosolic protein 2	LCP2	NM_005565	Tubules	Pneumocytes, macrophages
6	Lysosomal-associated membrane protein 3	LAMP3	NM_014398	N/A	Pneumocytes
7	Mucin 5B, oligomeric mucus/gel-forming	MUC5B	NM_002458	N/A	Epithelial cells of bronchus, nasopharynx, macrophages
8	Surfactant Protein B	SFTPB/ SP-B	NM_000542	N/A	Pneumocytes
9	Surfactant Protein C	SFTPC/ SP-C	NM_003018	Tubules	Pneumocytes, macrophages
10	Thyroid-specific transcription factor/NK2 homeobox 1	TTF1/ NKX2.1	NM_003317	N/A	Lung endoderm, pneumocytes, macrophages
11	Von Willebrand factor	VWF	NM_000552	N/A	Pneumocytes

N/A=not applicable
