# Supplementary material for: Tissue Specificity of Decellularized Rhesus Monkey Kidney and Lung Scaffolds
Source: PLoS One. 2013 May 22;8(5):e64134. doi: 10.1371/journal.pone.0064134 (PMC3661477; doi:10.1371/journal.pone.0064134)
Supplement: Table S4 — Protein Composition of Kidney and Lung Scaffolds (PDF) [file pone.0064134.s004.pdf]

Table S4. Protein Composition of Kidney and Lung Scaffolds

Accession #	Protein Name	Kidney Expression	Lung Expression
297285376	Actin, cytoplasmic 1-like isoform 1	H	H
297296560	Annexin A2 isoform 2	H	H
109132219	Apoptosis-inducing factor 1, mitochondrial-like isoform 2	VL	ND
297282427	Basement membrane-specific heparan sulfate proteoglycan core protein	M	H
109088110	Calmodulin-like 5	ND	VL
109123728	Caspase-14	VL	ND
47118104	CD36	ND	VL
355560218	Collagen alpha-1(VI) chain	M	H
109067475	Collagen alpha-2(I) chain isoform 3	M	M
297270263	Complement C5	L	ND
109077082	Complement component C6 isoform 2	L	ND
355691279	Complement component C7, partial	L	ND
109005039	Complement component C8 beta chain	L	ND
109077053	Complement component C9	H	VL
301129221	Cytochrome c oxidase subunit 6C	M	ND
302129663	Cytochrome c oxidase subunit IV isoform 1	L	ND
355568643	Cytokeratin-27	M	L
355564260	Cytokeratin-72	H	L
7993923	Defensin	ND	M
297262587	Dermcidin-like	M	L
302563337	Epidermal growth factor-like protein 7 precursor	VL	VL
307691215	Eukaryotic translation elongation factor 1	VL	L

	alpha 1		
109075963	Fibrinogen gamma chain isoform 9	M	M
297269442	Heat shock cognate 71 kDa protein isoform 4	VL	VL
306922404	Heat shock protein 90 kDa beta (Grp94), member 1 precursor	VL	ND
109066218	Heat shock protein beta-1	ND	VL
355566791	Hemoglobin beta chain	H	VH
109075615	Heparin-binding growth factor 2/fibroblast growth factor 2	ND	VL
109016355	Histone H2B 5-like	H	ND
34100900	Immunoglobulin gamma-2 heavy chain constant region	L	M
323404609	Immunoglobulin heavy chain	L	M
225625774	Immunoglobulin kappa light chain	L	L
66271935	Immunoglobulin light chain	ND	L
109074721	Insulin-like growth factor binding protein 7 isoform 4	VL	ND
297273003	Keratin, type I cytoskeletal 14-like isoform 1	VH	H
29726244	Keratin, type II cytoskeletal 1-like isoform 6	VH	VH
109096855	Keratin, type II cytoskeletal 8 isoform 1	H	ND
297259425	Laminin subunit alpha-5-like	L	M
109032913	Leukocyte surface antigen CD47 isoform 3	ND	L
109091737	Matrix metalloproteinase-9	ND	VL
355692973	Milk fat globule-EGF factor 8	ND	VL
109019927	Nidogen-1 isoform 2	M	M
297274297	Periostin	L	VH
109078702	Transforming growth factor-beta-induced protein ig-h3 isoform 6	VL	M

311213900	Ubiquinol-cytochrome c reductase, complex III subunit VII	M	ND
355698189	Undulin	VL	H
297283602	Uromodulin isoform 3	VL	ND
355562319	Vimentin	L	H
109113730	Vitronectin	M	L
108995558	Von Willebrand factor A domain-containing protein 1 isoform 2	ND	L

Expression of protein is based on the percent a given protein represents out of the total protein detected in the scaffold. Proteins were classified as very high (VH): >3%; high (H): 1-3%; middle (M): 0.5-1%; low (L): 0.1-0.5%; and very low (VL): <0.1% total protein. ND=Not detected
